# *catena*-Poly[[(1,10-phenanthroline-5,6-dione-κ^2^ *N*,*N*′)lead(II)]-μ-terephthalato-κ^2^ *O* ^1^:*O* ^4^]

**DOI:** 10.1107/S1600536809046455

**Published:** 2009-11-07

**Authors:** Fang-Di Cong, Feng-Yang Yu, Zhen Wei, Seik Weng Ng

**Affiliations:** a Department of Basic Science, Tianjin Agricultural University, Tjianjin 300384, People’s Republic of China; b Department of Chemistry, University of Malaya, 50603 Kuala Lumpur, Malaysia

## Abstract

The Pb^II^ atom in the polymeric title compound, [Pb(C_8_H_4_O_4_)(C_12_H_6_N_2_O_2_)]_*n*_, is chelated by the N-heterocycle, and adjacent atoms are bridged by rigid terephthalate dianions into a linear chain. The Pb^II^ atom is stereochemically active in a ψ-square-pyramidal coordination geometry in which the lone-pair electrons occupy a basal site. When three other weaker Pb⋯O inter­actions are considered, the geometry is a ψ-dodeca­hedron.

## Related literature

For the crystal structure of lead(II) terephthalate, see: Tan *et al.* (2009 ▶).
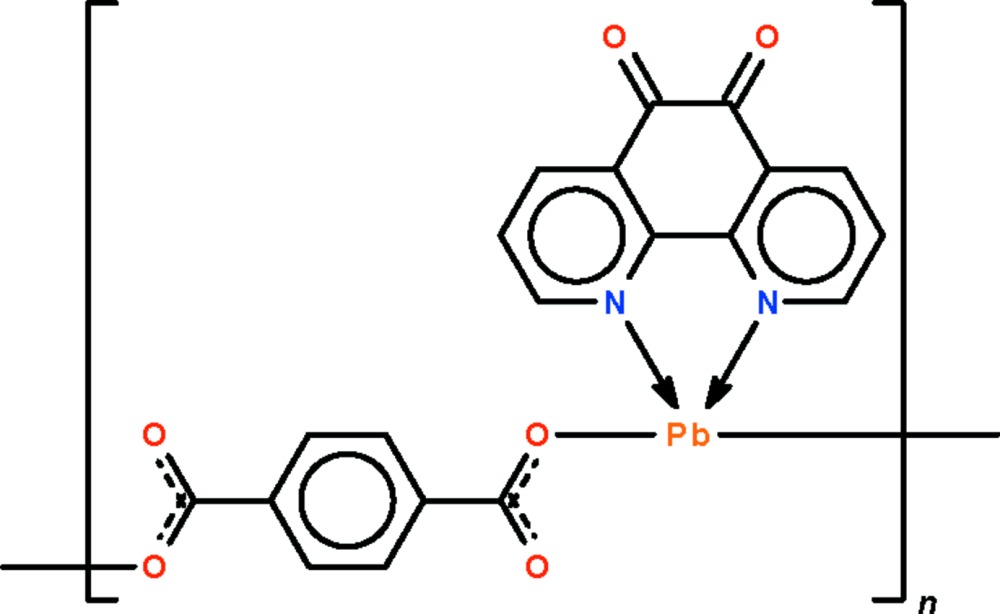


## Experimental

### 

#### Crystal data

[Pb(C_8_H_4_O_4_)(C_12_H_6_N_2_O_2_)]
*M* *_r_* = 581.49
Monoclinic, 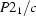

*a* = 10.428 (1) Å
*b* = 15.187 (1) Å
*c* = 11.478 (3) Å
β = 114.444 (1)°
*V* = 1654.8 (5) Å^3^
*Z* = 4
Mo *K*α radiation
μ = 10.24 mm^−1^
*T* = 295 K
0.24 × 0.22 × 0.20 mm

#### Data collection

Bruker APEXII area-detector diffractometer
Absorption correction: multi-scan (*SADABS*; Sheldrick, 1996 ▶) *T*_min_ = 0.193, *T*_max_ = 0.234
8875 measured reflections
3233 independent reflections
2369 reflections with *I* > 2σ(*I*)
*R*_int_ = 0.036

#### Refinement

*R*[*F* ^2^ > 2σ(*F* ^2^)] = 0.033
*wR*(*F* ^2^) = 0.086
*S* = 1.00
3233 reflections
262 parameters
H-atom parameters constrained
Δρ_max_ = 1.94 e Å^−3^
Δρ_min_ = −0.95 e Å^−3^

### 

Data collection: *APEX2* (Bruker, 2007 ▶); cell refinement: *SAINT* (Bruker, 2007 ▶); data reduction: *SAINT*; program(s) used to solve structure: *SHELXS97* (Sheldrick, 2008 ▶); program(s) used to refine structure: *SHELXL97* (Sheldrick, 2008 ▶); molecular graphics: *X-SEED* (Barbour, 2001 ▶); software used to prepare material for publication: *publCIF* (Westrip, 2009 ▶).

## Supplementary Material

Crystal structure: contains datablocks global, I. DOI: 10.1107/S1600536809046455/ci2964sup1.cif

Structure factors: contains datablocks I. DOI: 10.1107/S1600536809046455/ci2964Isup2.hkl

Additional supplementary materials:  crystallographic information; 3D view; checkCIF report

## Figures and Tables

**Table 1:** Selected bond lengths (Å)

Pb1—O1	2.410 (6)
Pb1—O2	3.211 (5)
Pb1—O2^i^	3.176 (6)
Pb1—O3^ii^	2.301 (5)
Pb1—O4^ii^	2.910 (5)
Pb1—N1	2.535 (6)
Pb1—N2	2.626 (6)

Symmetry codes: (i) 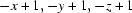; (ii) 
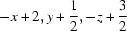
.

**Fig. 1.**
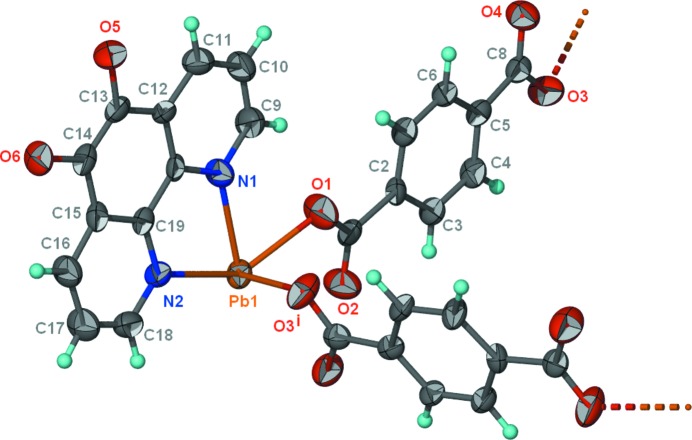
Displacement ellipsoid plot (Barbour, 2001) of a part of the polymeric Pb(C8H4O2)(C12H6N2O2) chain at the 70% probability level; H atoms are drawn as spheres of arbitrary radius. Symmetry code: (i) 2-x, 1/2+y, 3/2-z.

**Fig. 2.**
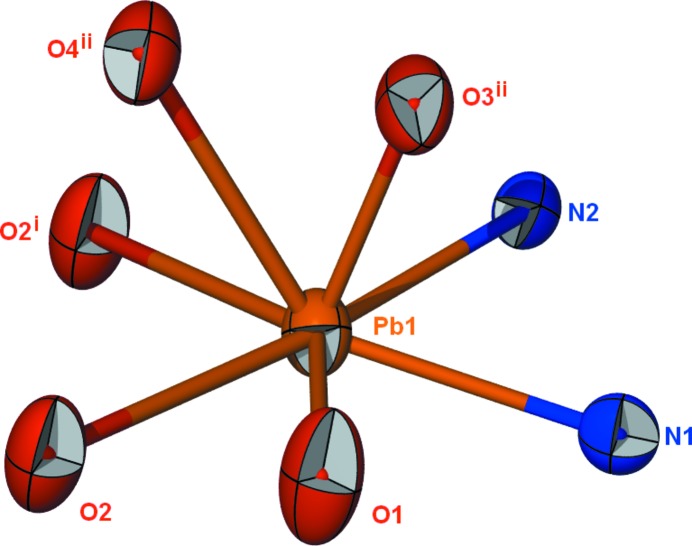
Detail of the polyhedron surrounding the lead atom. Symmetry codes: (i) 1-x, 1-y, 1-z; (ii) 2-x, 1/2+y, 3/2-z.

## supplementary crystallographic information

### Experimental

Lead nitrate (0.5 mmol, 0.166 g), terephthalic acid (0.5 mmol, 0.083 g) and phenanthrene-9,10-dione (0.5 mmol, 0.104 g) in water (12 ml) were heated at 443 K for 3 days in a Teflon-lined, stainless-steel Parr bomb. The bomb was slowly cooled to room temperature after which crystals were collected in 40% yield.

### Refinement

Carbon-bound H-atoms were placed in calculated positions (C-H = 0.93 Å) and were included in the refinement in the riding model approximation, with *U_iso_*(H) set to 1.2*U_eq_*(C). The final difference Fourier map had one peak in the vicinity of the lead atom (0.95 Å from Pb1) but was otherwise diffuse.

### Crystal data

**Table:**

[Pb(C_8_H_4_O_4_)(C_12_H_6_N_2_O_2_)]	*F*(000) = 1096
*M**_r_* = 581.49	*D*_x_ = 2.334 Mg m^−^^3^
Monoclinic, *P*2_1_/*c*	Mo *K*α radiation, λ = 0.71073 Å
Hall symbol: -P 2ybc	Cell parameters from 2807 reflections
*a* = 10.428 (1) Å	θ = 2.4–25.4°
*b* = 15.187 (1) Å	µ = 10.24 mm^−^^1^
*c* = 11.478 (3) Å	*T* = 295 K
β = 114.444 (1)°	Block, yellow
*V* = 1654.8 (5) Å^3^	0.24 × 0.22 × 0.20 mm
*Z* = 4	

### Data collection

**Table:**

Bruker APEXII area-detector diffractometer	3233 independent reflections
Radiation source: fine-focus sealed tube	2369 reflections with *I* > 2σ(*I*)
graphite	*R*_int_ = 0.036
φ and ω scans	θ_max_ = 26.0°, θ_min_ = 2.2°
Absorption correction: multi-scan (*SADABS*; Sheldrick, 1996)	*h* = −12→7
*T*_min_ = 0.193, *T*_max_ = 0.234	*k* = −18→18
8875 measured reflections	*l* = −12→14

### Refinement

**Table:**

Refinement on *F*^2^	Primary atom site location: structure-invariant direct methods
Least-squares matrix: full	Secondary atom site location: difference Fourier map
*R*[*F*^2^ > 2σ(*F*^2^)] = 0.033	Hydrogen site location: inferred from neighbouring sites
*wR*(*F*^2^) = 0.086	H-atom parameters constrained
*S* = 1.00	*w* = 1/[σ^2^(*F*_o_^2^) + (0.0491*P*)^2^] where *P* = (*F*_o_^2^ + 2*F*_c_^2^)/3
3233 reflections	(Δ/σ)_max_ = 0.001
262 parameters	Δρ_max_ = 1.94 e Å^−^^3^
0 restraints	Δρ_min_ = −0.95 e Å^−^^3^

### Fractional atomic coordinates and isotropic or equivalent isotropic displacement parameters (Å^2^)

**Table:**

	*x*	*y*	*z*	*U*_iso_*/*U*_eq_	
Pb1	0.60601 (3)	0.525108 (19)	0.39442 (2)	0.02978 (11)	
O1	0.8123 (7)	0.4460 (4)	0.5353 (5)	0.0548 (17)	
O2	0.7155 (6)	0.4344 (4)	0.6715 (5)	0.0489 (15)	
O3	1.2513 (6)	0.1473 (4)	1.0397 (5)	0.0434 (14)	
O4	1.3459 (6)	0.1510 (4)	0.9005 (5)	0.0414 (14)	
O5	0.7884 (6)	0.5551 (4)	−0.1400 (5)	0.0396 (14)	
O6	0.5419 (6)	0.6582 (4)	−0.2269 (4)	0.0401 (14)	
N1	0.7358 (7)	0.4976 (4)	0.2537 (6)	0.0313 (15)	
N2	0.5139 (6)	0.6109 (4)	0.1764 (5)	0.0273 (14)	
C1	0.8093 (8)	0.4176 (5)	0.6363 (6)	0.0274 (16)	
C2	0.9298 (7)	0.3592 (5)	0.7180 (6)	0.0269 (16)	
C3	0.9398 (8)	0.3284 (5)	0.8348 (6)	0.0328 (18)	
H3	0.8732	0.3456	0.8647	0.039*	
C4	1.0481 (8)	0.2719 (5)	0.9082 (6)	0.0307 (17)	
H4	1.0530	0.2514	0.9862	0.037*	
C5	1.1492 (8)	0.2459 (4)	0.8653 (6)	0.0250 (15)	
C6	1.1423 (8)	0.2799 (5)	0.7518 (6)	0.0266 (16)	
H6	1.2107	0.2642	0.7234	0.032*	
C7	1.0367 (8)	0.3362 (4)	0.6798 (6)	0.0279 (17)	
H7	1.0359	0.3595	0.6046	0.033*	
C8	1.2578 (8)	0.1772 (5)	0.9386 (7)	0.0274 (16)	
C9	0.8489 (9)	0.4464 (5)	0.2881 (7)	0.039 (2)	
H9	0.8807	0.4198	0.3681	0.047*	
C10	0.9226 (8)	0.4296 (6)	0.2157 (7)	0.0373 (19)	
H10	1.0007	0.3927	0.2460	0.045*	
C11	0.8796 (8)	0.4678 (5)	0.0993 (7)	0.0353 (18)	
H11	0.9284	0.4576	0.0487	0.042*	
C12	0.7620 (7)	0.5226 (5)	0.0555 (6)	0.0253 (15)	
C13	0.7110 (8)	0.5659 (5)	−0.0684 (6)	0.0291 (17)	
C14	0.5958 (8)	0.6182 (5)	−0.1084 (6)	0.0294 (16)	
C15	0.5252 (8)	0.6348 (5)	−0.0279 (6)	0.0275 (16)	
C16	0.4105 (8)	0.6930 (5)	−0.0603 (7)	0.0371 (19)	
H16	0.3745	0.7208	−0.1397	0.044*	
C17	0.3523 (9)	0.7087 (5)	0.0235 (7)	0.041 (2)	
H17	0.2778	0.7481	0.0027	0.049*	
C18	0.4045 (8)	0.6657 (5)	0.1401 (7)	0.0354 (19)	
H18	0.3614	0.6754	0.1956	0.042*	
C19	0.5741 (7)	0.5947 (4)	0.0952 (6)	0.0230 (15)	
C20	0.6907 (7)	0.5364 (4)	0.1342 (6)	0.0237 (15)	

### Atomic displacement parameters (Å^2^)

**Table:**

	*U*^11^	*U*^22^	*U*^33^	*U*^12^	*U*^13^	*U*^23^
Pb1	0.02839 (17)	0.03876 (18)	0.02215 (15)	−0.00225 (15)	0.01043 (12)	0.00065 (13)
O1	0.044 (4)	0.090 (5)	0.032 (3)	0.022 (3)	0.018 (3)	0.020 (3)
O2	0.045 (4)	0.067 (4)	0.043 (3)	0.017 (3)	0.026 (3)	0.012 (3)
O3	0.054 (4)	0.050 (3)	0.032 (3)	0.017 (3)	0.024 (3)	0.012 (3)
O4	0.039 (3)	0.055 (4)	0.035 (3)	0.011 (3)	0.020 (3)	0.009 (3)
O5	0.037 (3)	0.060 (4)	0.031 (3)	0.000 (3)	0.025 (3)	0.000 (3)
O6	0.040 (3)	0.058 (4)	0.022 (3)	0.007 (3)	0.012 (3)	0.012 (2)
N1	0.031 (4)	0.031 (3)	0.030 (3)	0.007 (3)	0.012 (3)	0.007 (2)
N2	0.029 (4)	0.030 (3)	0.024 (3)	0.000 (3)	0.013 (3)	−0.001 (2)
C1	0.030 (4)	0.031 (4)	0.020 (4)	−0.003 (3)	0.010 (3)	−0.005 (3)
C2	0.023 (4)	0.031 (4)	0.020 (4)	0.000 (3)	0.002 (3)	−0.001 (3)
C3	0.032 (4)	0.048 (5)	0.019 (4)	0.006 (4)	0.011 (3)	0.002 (3)
C4	0.035 (4)	0.038 (4)	0.019 (4)	0.001 (4)	0.011 (3)	0.007 (3)
C5	0.030 (4)	0.023 (4)	0.023 (4)	−0.005 (3)	0.012 (3)	−0.004 (3)
C6	0.028 (4)	0.036 (4)	0.019 (3)	0.001 (3)	0.013 (3)	0.001 (3)
C7	0.032 (4)	0.030 (4)	0.021 (4)	0.000 (3)	0.011 (3)	0.004 (3)
C8	0.028 (4)	0.029 (4)	0.025 (4)	−0.005 (3)	0.012 (3)	−0.004 (3)
C9	0.042 (5)	0.044 (5)	0.028 (4)	0.013 (4)	0.010 (4)	0.005 (3)
C10	0.031 (4)	0.049 (5)	0.031 (4)	0.007 (4)	0.012 (4)	0.006 (4)
C11	0.035 (5)	0.038 (4)	0.038 (4)	0.001 (4)	0.020 (4)	−0.003 (4)
C12	0.024 (4)	0.031 (4)	0.022 (3)	0.000 (4)	0.011 (3)	−0.007 (3)
C13	0.030 (4)	0.035 (4)	0.023 (4)	−0.005 (4)	0.012 (3)	−0.010 (3)
C14	0.031 (4)	0.035 (4)	0.020 (3)	−0.009 (4)	0.009 (3)	−0.002 (3)
C15	0.023 (4)	0.034 (4)	0.021 (4)	−0.005 (3)	0.005 (3)	−0.001 (3)
C16	0.034 (5)	0.043 (5)	0.031 (4)	0.008 (4)	0.010 (4)	0.008 (4)
C17	0.041 (5)	0.042 (5)	0.036 (5)	0.011 (4)	0.014 (4)	0.012 (4)
C18	0.033 (5)	0.038 (4)	0.037 (5)	0.002 (4)	0.016 (4)	−0.008 (3)
C19	0.024 (4)	0.023 (3)	0.020 (3)	−0.007 (3)	0.007 (3)	−0.003 (3)
C20	0.020 (4)	0.030 (4)	0.017 (3)	−0.005 (3)	0.002 (3)	−0.005 (3)

### Geometric parameters (Å, °)

**Table:**

Pb1—O1	2.410 (6)	C5—C6	1.375 (9)
Pb1—O2	3.211 (5)	C5—C8	1.514 (10)
Pb1—O2^i^	3.176 (6)	C6—C7	1.369 (9)
Pb1—O3^ii^	2.301 (5)	C6—H6	0.93
Pb1—O4^ii^	2.910 (5)	C7—H7	0.93
Pb1—N1	2.535 (6)	C9—C10	1.369 (11)
Pb1—N2	2.626 (6)	C9—H9	0.93
O1—C1	1.249 (8)	C10—C11	1.352 (10)
O2—C1	1.230 (9)	C10—H10	0.93
O3—C8	1.273 (8)	C11—C12	1.392 (10)
O4—C8	1.235 (9)	C11—H11	0.93
O5—C13	1.380 (8)	C12—C20	1.403 (9)
O6—C14	1.379 (8)	C12—C13	1.453 (10)
N1—C9	1.329 (10)	C13—C14	1.352 (10)
N1—C20	1.384 (9)	C14—C15	1.423 (10)
N2—C18	1.332 (9)	C15—C16	1.408 (10)
N2—C19	1.345 (8)	C15—C19	1.425 (9)
C1—C2	1.507 (10)	C16—C17	1.354 (10)
C2—C3	1.382 (9)	C16—H16	0.93
C2—C7	1.401 (10)	C17—C18	1.383 (10)
C3—C4	1.390 (10)	C17—H17	0.93
C3—H3	0.93	C18—H18	0.93
C4—C5	1.392 (10)	C19—C20	1.419 (9)
C4—H4	0.93		
			
O3^ii^—Pb1—O1	84.5 (2)	C7—C6—C5	121.3 (7)
O3^ii^—Pb1—N1	84.2 (2)	C7—C6—H6	119.3
O1—Pb1—N1	77.40 (19)	C5—C6—H6	119.3
O3^ii^—Pb1—N2	81.07 (18)	C6—C7—C2	120.8 (6)
O1—Pb1—N2	139.46 (19)	C6—C7—H7	119.6
N1—Pb1—N2	63.61 (19)	C2—C7—H7	119.6
O3^ii^—Pb1—O4^ii^	48.16 (16)	O4—C8—O3	122.4 (7)
O1—Pb1—O4^ii^	88.69 (19)	O4—C8—C5	121.3 (6)
N1—Pb1—O4^ii^	131.65 (18)	O3—C8—C5	116.3 (7)
N2—Pb1—O4^ii^	108.50 (17)	N1—C9—C10	125.5 (7)
O3^ii^—Pb1—O2^i^	113.41 (19)	N1—C9—H9	117.2
O1—Pb1—O2^i^	144.83 (17)	C10—C9—H9	117.2
N1—Pb1—O2^i^	132.01 (18)	C11—C10—C9	118.7 (8)
N2—Pb1—O2^i^	75.11 (16)	C11—C10—H10	120.7
O4^ii^—Pb1—O2^i^	82.96 (15)	C9—C10—H10	120.7
O3^ii^—Pb1—O2	95.96 (17)	C10—C11—C12	119.7 (7)
O1—Pb1—O2	43.00 (17)	C10—C11—H11	120.1
N1—Pb1—O2	119.73 (17)	C12—C11—H11	120.1
N2—Pb1—O2	175.42 (16)	C11—C12—C20	118.6 (6)
O4^ii^—Pb1—O2	66.98 (16)	C11—C12—C13	122.4 (6)
O2^i^—Pb1—O2	103.09 (13)	C20—C12—C13	119.0 (6)
C1—O1—Pb1	115.9 (5)	C14—C13—O5	121.6 (7)
C1—O2—Pb1	76.6 (4)	C14—C13—C12	120.9 (6)
C8—O3—Pb1^iii^	108.4 (5)	O5—C13—C12	117.5 (6)
C9—N1—C20	116.0 (7)	C13—C14—O6	122.4 (7)
C9—N1—Pb1	123.3 (5)	C13—C14—C15	120.6 (7)
C20—N1—Pb1	120.6 (5)	O6—C14—C15	117.0 (7)
C18—N2—C19	118.9 (6)	C16—C15—C14	123.4 (7)
C18—N2—Pb1	122.4 (5)	C16—C15—C19	116.6 (7)
C19—N2—Pb1	118.6 (4)	C14—C15—C19	119.9 (7)
O2—C1—O1	124.2 (7)	C17—C16—C15	120.3 (7)
O2—C1—C2	119.3 (6)	C17—C16—H16	119.9
O1—C1—C2	116.5 (7)	C15—C16—H16	119.9
C3—C2—C7	117.9 (7)	C16—C17—C18	119.5 (8)
C3—C2—C1	120.8 (7)	C16—C17—H17	120.3
C7—C2—C1	121.2 (6)	C18—C17—H17	120.3
C2—C3—C4	120.8 (7)	N2—C18—C17	122.7 (7)
C2—C3—H3	119.6	N2—C18—H18	118.7
C4—C3—H3	119.6	C17—C18—H18	118.7
C3—C4—C5	120.4 (6)	N2—C19—C20	118.7 (6)
C3—C4—H4	119.8	N2—C19—C15	122.0 (6)
C5—C4—H4	119.8	C20—C19—C15	119.2 (6)
C6—C5—C4	118.5 (7)	N1—C20—C12	121.4 (6)
C6—C5—C8	121.3 (7)	N1—C20—C19	118.3 (6)
C4—C5—C8	120.1 (6)	C12—C20—C19	120.2 (6)
			
O3^ii^—Pb1—O1—C1	108.0 (6)	Pb1^iii^—O3—C8—O4	−10.9 (9)
N1—Pb1—O1—C1	−166.8 (6)	Pb1^iii^—O3—C8—C5	167.9 (5)
N2—Pb1—O1—C1	177.4 (5)	C6—C5—C8—O4	1.9 (11)
O4^ii^—Pb1—O1—C1	59.9 (6)	C4—C5—C8—O4	177.7 (7)
O2^i^—Pb1—O1—C1	−15.9 (8)	C6—C5—C8—O3	−177.0 (7)
O2—Pb1—O1—C1	3.2 (5)	C4—C5—C8—O3	−1.2 (10)
O3^ii^—Pb1—O2—C1	−78.4 (5)	C20—N1—C9—C10	0.1 (12)
O1—Pb1—O2—C1	−3.0 (5)	Pb1—N1—C9—C10	−179.9 (7)
N1—Pb1—O2—C1	8.3 (5)	N1—C9—C10—C11	0.4 (13)
O4^ii^—Pb1—O2—C1	−117.8 (5)	C9—C10—C11—C12	−0.3 (12)
O2^i^—Pb1—O2—C1	165.8 (5)	C10—C11—C12—C20	−0.2 (11)
O3^ii^—Pb1—N1—C9	93.9 (6)	C10—C11—C12—C13	179.7 (7)
O1—Pb1—N1—C9	8.2 (6)	C11—C12—C13—C14	179.5 (7)
N2—Pb1—N1—C9	176.8 (7)	C20—C12—C13—C14	−0.6 (11)
O4^ii^—Pb1—N1—C9	84.9 (6)	C11—C12—C13—O5	−3.7 (10)
O2^i^—Pb1—N1—C9	−149.6 (6)	C20—C12—C13—O5	176.2 (6)
O2—Pb1—N1—C9	0.3 (7)	O5—C13—C14—O6	5.3 (11)
O3^ii^—Pb1—N1—C20	−86.2 (5)	C12—C13—C14—O6	−178.0 (6)
O1—Pb1—N1—C20	−171.9 (6)	O5—C13—C14—C15	−174.1 (6)
N2—Pb1—N1—C20	−3.3 (5)	C12—C13—C14—C15	2.6 (11)
O4^ii^—Pb1—N1—C20	−95.2 (5)	C13—C14—C15—C16	175.8 (7)
O2^i^—Pb1—N1—C20	30.3 (6)	O6—C14—C15—C16	−3.7 (11)
O2—Pb1—N1—C20	−179.7 (5)	C13—C14—C15—C19	−1.4 (11)
O3^ii^—Pb1—N2—C18	−92.5 (6)	O6—C14—C15—C19	179.2 (6)
O1—Pb1—N2—C18	−163.1 (5)	C14—C15—C16—C17	−177.0 (8)
N1—Pb1—N2—C18	179.6 (6)	C19—C15—C16—C17	0.2 (11)
O4^ii^—Pb1—N2—C18	−52.4 (6)	C15—C16—C17—C18	−1.4 (13)
O2^i^—Pb1—N2—C18	24.8 (5)	C19—N2—C18—C17	−1.9 (11)
O3^ii^—Pb1—N2—C19	91.7 (5)	Pb1—N2—C18—C17	−177.6 (6)
O1—Pb1—N2—C19	21.1 (6)	C16—C17—C18—N2	2.3 (13)
N1—Pb1—N2—C19	3.8 (5)	C18—N2—C19—C20	180.0 (6)
O4^ii^—Pb1—N2—C19	131.8 (5)	Pb1—N2—C19—C20	−4.1 (8)
O2^i^—Pb1—N2—C19	−151.0 (5)	C18—N2—C19—C15	0.5 (10)
Pb1—O2—C1—O1	4.8 (7)	Pb1—N2—C19—C15	176.5 (5)
Pb1—O2—C1—C2	−175.3 (6)	C16—C15—C19—N2	0.3 (10)
Pb1—O1—C1—O2	−6.9 (10)	C14—C15—C19—N2	177.6 (6)
Pb1—O1—C1—C2	173.2 (5)	C16—C15—C19—C20	−179.1 (6)
O2—C1—C2—C3	−3.5 (11)	C14—C15—C19—C20	−1.8 (10)
O1—C1—C2—C3	176.5 (7)	C9—N1—C20—C12	−0.7 (10)
O2—C1—C2—C7	178.1 (7)	Pb1—N1—C20—C12	179.4 (5)
O1—C1—C2—C7	−1.9 (10)	C9—N1—C20—C19	−177.3 (7)
C7—C2—C3—C4	−3.9 (11)	Pb1—N1—C20—C19	2.7 (8)
C1—C2—C3—C4	177.6 (7)	C11—C12—C20—N1	0.7 (10)
C2—C3—C4—C5	0.5 (11)	C13—C12—C20—N1	−179.2 (6)
C3—C4—C5—C6	2.4 (10)	C11—C12—C20—C19	177.3 (6)
C3—C4—C5—C8	−173.6 (7)	C13—C12—C20—C19	−2.6 (10)
C4—C5—C6—C7	−1.7 (10)	N2—C19—C20—N1	1.0 (9)
C8—C5—C6—C7	174.2 (6)	C15—C19—C20—N1	−179.5 (6)
C5—C6—C7—C2	−1.8 (11)	N2—C19—C20—C12	−175.7 (6)
C3—C2—C7—C6	4.6 (11)	C15—C19—C20—C12	3.8 (9)
C1—C2—C7—C6	−177.0 (6)		

Symmetry codes: (i) −*x*+1, −*y*+1, −*z*+1; (ii) −*x*+2, *y*+1/2, −*z*+3/2; (iii) −*x*+2, *y*−1/2, −*z*+3/2.
